# Genome Sequence of “*Candidatus* Methanomassiliicoccus intestinalis” Issoire-Mx1, a Third *Thermoplasmatales*-Related Methanogenic Archaeon from Human Feces

**DOI:** 10.1128/genomeA.00453-13

**Published:** 2013-07-11

**Authors:** Guillaume Borrel, Hugh M. B. Harris, Nicolas Parisot, Nadia Gaci, William Tottey, Agnès Mihajlovski, Jennifer Deane, Simonetta Gribaldo, Olivier Bardot, Eric Peyretaillade, Pierre Peyret, Paul W. O’Toole, Jean-François Brugère

**Affiliations:** EA-4678 CIDAM, Conception Ingénierie et Développement de l’Aliment et du Médicament, Clermont Université, Université d’Auvergne, Clermont-Ferrand, Francea; Department of Microbiology and Alimentary Pharmabiotic Centre, University College Cork, Cork, Irelandb; Unité de Biologie Moléculaire du Gène chez les Extrêmophiles, Institut Pasteur, Paris, Francec; GReD, CNRS UMR 6923, INSERM UMR 1103, Clermont Université, Université d’Auvergne, Clermont-Ferrand, Franced

## Abstract

“*Candidatus* Methanomassiliicoccus intestinalis” Issoire-Mx1 is a methanogenic archaeon found in the human gut and is a representative of the novel order of methanogens related to *Thermoplasmatales*. Its complete genome sequence is presented here.

## GENOME ANNOUNCEMENT

In recent years, growing evidence from culture-independent studies has suggested that methanogens associated with the human gut are far more diverse than was thought (1–4) and partly belong to a *Thermoplasmatales*-related lineage (2). The methanogenic nature of this lineage was confirmed by the description of *Methanomassiliicoccus luminyensis* strain B10, isolated from human feces (5). Another methanogen of this *Thermoplasmatales*-related lineage, “*Candidatus* Methanomethylophilus alvus” Mx1201, was also cultured from human feces and was found to be distantly related to *M. luminyensis*. The genomes of both were recently sequenced (6, 7).

A highly enriched culture of “*Candidatus* Methanomassiliicoccus intestinalis” Issoire-Mx1 was obtained by the same procedure followed for “*Ca*. Methanomethylophilus alvus” (6). This archaeon is distantly related to “*Ca*. Methanomethylophilus alvus” and is closely related to *M. luminyensis*, with 87% and 98% of 16S rRNA gene sequence identity, respectively. The affiliation of *M. luminyensis* and “*Ca*. Methanomassiliicoccus intestinalis” to a large cluster of sequences retrieved from paddy soils and freshwater and marine sediments suggests their recent adaptation to gut environments.

A 3-kb mate-paired library was constructed and sequenced from a quarter plate of a 454 GS FLX Titanium run (Macrogen, Republic of Korea). A total of 283,279 reads corresponding to 125.5 Mb were obtained. The reads were assembled with Newbler (v2.3), first in 28 contigs (average depth coverage of 42.7-fold) and then in a unique scaffold. The gaps between the contigs were closed by sequencing. The open reading frames were predicted with Glimmer 3 (8), were annotated using RAST (9), and were manually curated.

“*Ca*. Methanomassiliicoccus intestinalis” has a circular genome of 1,931,561 bp, with a G+C content of 41.3%. Despite its close phylogenetic relationship with *M. luminyensis*, the genome of “*Ca*. Methanomassiliicoccus intestinalis” is 27% smaller and its G+C content is 20% lower than that of *M. luminyensis*. This suggests a fast genomic reshuffle in one of the two genomes, which may be due to differential adaptation to the gut environment. The “*Ca*. Methanomassiliicoccus intestinalis” genome contains 46 tRNA genes, a single copy of the 16S and 23S rRNA genes, and 2 noncontiguous copies of 5S rRNA genes that were distant from the 23S and 16S rRNA genes. A total of 1,820 protein-coding sequences were predicted. A clustered regularly interspaced short palindromic repeat (CRISPR) region containing 110 spacers was identified using CRISPRfinder (10), in close association with *cas* genes. The genome of “*Ca*. Methanomassiliicoccus intestinalis” contains one *mcr* operon (*mcrBDGA*) and a *mcrC* gene distantly located from it. Genes involved in methylotrophic methanogenesis from methanol (*mtaABC*) and methylamine compounds (*mtmBC*, *mtbBC*, and *mttBC*) are also present. The latter are on an 18.7-kb region also containing the genes involved in pyrrolysine biosynthesis. The sequence of “*Ca*. Methanomassiliicoccus intestinalis” offers a great opportunity to determine the metabolic properties and phenotypic features of this poorly characterized order of methanogens related to *Thermoplasmatales*, and it will further help to identify the genomic adaptations of methanogens to gut environments.

### Nucleotide sequence accession number.

The draft genome sequence of “*Candidatus* Methanomassiliicoccus intestinalis” Issoire-Mx1 has been deposited in GenBank under the accession no. CP005934.
